# Wzx flippases exhibiting complex O‐unit preferences require a new model for Wzx–substrate interactions

**DOI:** 10.1002/mbo3.655

**Published:** 2018-06-10

**Authors:** Michael A. Liu, Paraskevi Morris, Peter R. Reeves

**Affiliations:** ^1^ School of Life and Environmental Sciences The University of Sydney Sydney New South Wales Australia

**Keywords:** flippase, lipopolysaccharide, O antigen, specificity, Wzx

## Abstract

The Wzx flippase is a critical component of the O‐antigen biosynthesis pathway, being responsible for the translocation of oligosaccharide O units across the inner membrane in Gram‐negative bacteria. Recent studies have shown that Wzx has a strong preference for its cognate O unit, but the types of O‐unit structural variance that a given Wzx can accommodate are poorly understood. In this study, we identified two *Yersinia pseudotuberculosis* Wzx that can distinguish between different terminal dideoxyhexose sugars on a common O‐unit main‐chain, despite both being able to translocate several other structurally‐divergent O units. We also identified other *Y. pseudotuberculosis* Wzx that can translocate a structurally divergent foreign O unit with high efficiency, and thus exhibit an apparently relaxed substrate preference. It now appears that Wzx substrate preference is more complex than previously suggested, and that not all O‐unit residues are equally important determinants of translocation efficiency. We propose a new “Structure‐Specific Triggering” model in which Wzx translocation proceeds at a low level for a wide variety of substrates, with high‐frequency translocation only being triggered by Wzx interacting with one or more preferred O‐unit structural elements found on its cognate O unit(s).

## 1 INTRODUCTION

The O‐antigen component of lipopolysaccharide (LPS) is the outermost cell component in many Gram‐negative bacteria, and plays an important protective role that is best understood for pathogens (Joiner, 1988; Matamouros & Miller, 2015; Murray, Attridge, & Morona, 2006). O antigens consist of short repeating oligosaccharide units (O units), each with typically 3–8 sugars, which can vary enormously in composition, even within a species. For instance, there are at least 185 currently identified O antigens in *Escherichia coli* (DebRoy et al., 2016), 46 in *Salmonella enterica* (Liu et al., 2014) and 34 in *Shigella* (Liu et al., 2008). This diversity is likely driven by selective pressures arising from the highly antigenic nature of O antigen as a component of LPS.

The vast majority of heteropolymeric O antigens are synthesized via the Wzx/Wzy pathway (Kalynych, Morona, & Cygler, 2014; Reeves & Cunneen, 2009), with most of the responsible genes grouped together in a gene cluster at a conserved chromosomal location. O‐unit synthesis is initiated at the cytoplasmic face of the inner membrane (IM), where a phosphoglycosyl transferase attaches the first sugar phosphate (typically *N*‐acetylglucosamine phosphate (GlcNAc‐P) in the Enterobacteriaceae) onto the polyisoprenoid lipid carrier molecule undecaprenyl phosphate (UndP), generating a pyrophosphate (PP) linkage (Price & Momany, 2005). A series of glycosyltransferases (GTs) then sequentially add the remaining sugars onto the growing chain to form a complete UndPP‐linked O unit. The Wzx flippase then translocates these UndPP‐linked O units across the IM to the periplasmic face, where the Wzy polymerase assembles polymeric UndPP‐linked O antigen under the control of the chain‐length regulator Wzz. The WaaL ligase then removes the O antigen from UndPP and attaches it to the lipid A‐core component of LPS, which is synthesized separately (Raetz & Whitfield, 2002), and the complete LPS molecule is exported to the outer leaflet of the outer membrane via the Lpt pathway (Whitfield & Trent, 2014).

Wzx was first proposed to be the O‐unit flippase over 20 years ago (Liu, Cole, & Reeves, 1996), and this is now generally accepted given the absence of a credible alternative flippase candidate (Hong, Liu, & Reeves, 2018; Islam & Lam, 2013). While there is data supporting the role of Wzx in O‐unit translocation (Rick et al., 2003), definitive biochemical proof remains elusive. Wzx has long been overlooked as a key factor in the evolution of O‐antigen diversity, largely due to early studies concluding that despite the enormous amount of sequence diversity among different Wzx, they were specific only for the first O‐unit residue (Feldman et al., 1999; Marolda, Vicarioli, & Valvano, 2004). However it has since been demonstrated on several occasions that Wzx can exhibit a strong preference for residues beyond the first sugar (Hong et al., 2018). Furthermore, inefficient Wzx translocation of nonpreferred O‐unit substrates results in cytoplasmic O‐unit accumulation and sequestration of UndP, which in turn interferes with peptidoglycan synthesis, causing a loss of membrane integrity and ultimately cell lysis (Jorgenson & Young, 2016; Liu, Stent, Hong, & Reeves, 2015). It is now becoming clear that Wzx substrate specificity represents a key checkpoint in the O‐antigen biosynthesis pathway. In spite of this, our knowledge of the types of O‐unit structural features that can influence Wzx translocation efficiency are currently limited to the identity of the first sugar (Marolda et al., 2004) and the presence or absence of terminal side‐branch residues (Hong & Reeves, 2014; Liu et al., 2015).

**Figure 1:**
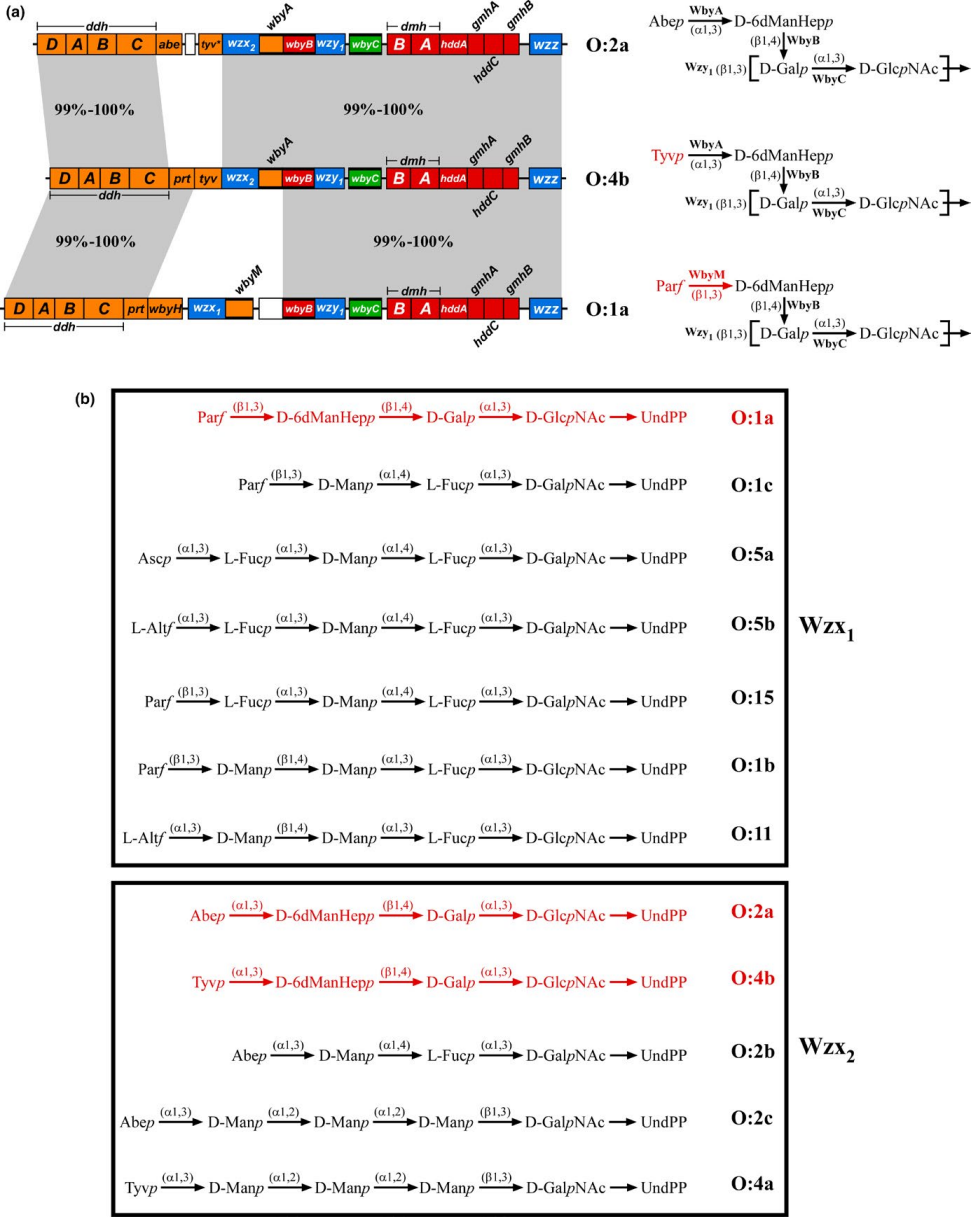
(a) The *Y. pseudotuberculosis* O:1a, O:2a and O:4b O‐antigen gene clusters (Pacinelli, Wang, & Reeves, 2002) and O‐unit structures (Kondakova et al., 2008, 2009, 2012). Gene clusters are drawn to scale on the left using the sequences from GenBank entries AF461768 to AF461770. Precursor sugar synthesis and glycosyltransferase (GT) genes are color‐coded based on their respective products: orange for the CDP‐dideoxyhexoses, red for CDP‐6‐deoxymannoheptose and green for UDP‐galactose, with the GT genes also indicated by bolded lines. The O‐antigen processing genes (*wzx*,* wzy* and *wzz*) are shown in blue, while remnant insertion sequence elements are indicated by the white boxes. Regions with high‐level nucleotide sequence identity (99–100%) between the gene clusters are shown within the gray shaded areas. The corresponding O‐unit structures are shown to the right of each gene cluster, along with the GTs responsible for each linkage. The square brackets denote the sites of the Wzy polymerization linkage. Residues, linkages and GTs on the O:1a and O:4b O units that differ from the O:2a O unit are shown in red. This figure is adapted from Kenyon et al. (2017). (b) Full range of *Y. pseudotuberculosis* O units translocated by the Wzx_1_ and Wzx_2_ flippases. O‐unit structures are shown without their polymerization linkages, as presented to Wzx prior to translocation. O units translocated by the same Wzx are grouped together into boxes, with the relevant Wzx indicated next to each box. The O units used in this study are shown in red

A group of 14 closely‐related *Yersinia pseudotuberculosis* O‐antigen gene clusters with four unique *wzx* sequence types, which produce O units with one of six alternative main‐chains and one of seven alternative side‐branch residues (Kenyon, Cunneen, & Reeves, 2017), represent a rare opportunity to expand our understanding of Wzx substrate preference. Within this set, the O:1a, O:2a, and O:4b O units (Figure 1a) consist of three shared residues (GlcNAc, galactose (Gal) and 6‐deoxymannoheptose (6dManHep)) with identical linkages, and one of three different terminal dideoxyhexose (DDH) residues (paratofuranose (Par*f*), O:1a; abequose (Abe*p*), O:2a; tyvelose (Tyv*p*), O:4b) attached via an α(1,3) (O:2a, O:4b) or β(1,3) (O:1a) linkage by different GTs (WbyA, O:2a/O:4b; WbyM, O:1a). All three O antigens share a common Wzy and polymerization linkage, where the 6dManHep and DDH residues become two‐sugar side‐branches. The corresponding O‐antigen gene clusters (Figure 1a) differ only in the genes responsible for DDH synthesis and attachment, and interestingly, contain one of two unrelated *wzx* genes (*wzx*_*1*_, O:1a; *wzx*_*2*_, O:2a/O:4b). These *wzx* are notably found in 12 of the 14 related gene clusters, and are exclusively associated with specific terminal DDH or DDH‐like residues (altrofuranose (Alt*f*), Par*f* or ascarylose (Asc*p*) with *wzx*_*1*_; Abe*p* or Tyv*p* with *wzx*_*2*_) on different O‐unit main‐chains (two main‐chains associated with both *wzx*, three main‐chains associated with only one *wzx*) (Kenyon et al., 2017) (Figure 1b). In this study we describe the cloning and manipulation of the *Y. pseudotuberculosis* O:1a, O:2a and O:4b O‐antigen gene clusters for expression in *E. coli*, and the use of these clones to conclusively demonstrate that both Wzx_1_ and Wzx_2_ show a strong preference for specific terminal DDH residues. Since both Wzx have several cognate O‐unit main‐chain structures, and several other *Y. pseudotuberculosis* Wzx are also shown to efficiently flip a foreign O unit in this study, we propose a new model for Wzx substrate preference by “Structure‐Specific Triggering” involving direct recognition of a small subset of O‐unit structural elements.

## 2 EXPERIMENTAL PROCEDURES

### 2.1 Strains and plasmids

The strains and plasmids used in this study are described in Table 1 and Table 2, respectively. *E. coli* and *Y. pseudotuberculosis* strains were grown in nutrient broth (10 g/L peptone, 5 g/L yeast extract, 5 g/L NaCl) at 37°C or 25°C, respectively. Gal‐free defined M9 media (6 g/L Na_2_HPO_4_, 3 g/L KH_2_PO_4_, 0.5 g/L NaCl, 1 g/L NH_4_Cl, 0.1 mM CaCl_2_, 1.6 mM MgSO_4_, 0.4% (v/v) glycerol, 20 mM sodium pyruvate, 3 g/L casamino acids, 8 μg/ml tryptophan, 1 μg/ml thiamine) was used when exogenous Gal was required to activate O‐antigen production. Agar was added (15 g/L) for solid growth media where required. Ampicillin (100 μg/ml), chloramphenicol (3 or 12.5 μg/ml), kanamycin (25 μg/ml) and/or streptomycin (1.6 mg/ml) were added as required for selection. *S. cerevisiae* strains were grown at 30°C in YPD broth (20 g/L peptone, 10 g/L yeast extract, 20 g/L glucose) or SD Ura^˗^ broth (6.7 g/L yeast nitrogen base (Sigma‐Aldrich), 1.92 g/L dropout supplement without uracil (Sigma‐Aldrich), 20 g/L glucose), with agar added (20 g/L) for solid growth media. Cycloheximide (2.5 μg/ml) was added to SD Ura^˗^ media for recombinant clone selection.

**Table 1:** Strains used in this study

Strain	Description	Source/Reference
CRY1‐2	*S. cerevisiae* recombinational cloning strain; *MAT*α, *ura3*Δ, *cyh2* ^*R*^	Raymond, Sims, and Olson (2002)
M85	*Y. pseudotuberculosis* serotype O:2a	Kessler et al. (1991)
M444	*Y. pseudotuberculosis* strain H892/87; serotype O:1a	Kessler, Haase, and Reeves (1993)
M451	*Y. pseudotuberculosis* strain H713/86; serotype O:3	Kessler et al. (1993)
M454	*Y. pseudotuberculosis* strain H715/56; serotype O:4b	Kessler et al. (1993)
M460	*Y. pseudotuberculosis* strain H720/86; serotype O:6	Kessler et al. (1993)
M462	*Y. pseudotuberculosis* strain H455/86; serotype O:7	Kessler et al. (1993)
M2575	*Y. pseudotuberculosis* strain 6088; serotype O:10	Kenyon et al. (2011)
M2577	*Y. pseudotuberculosis* strain MW864‐2; serotype O:12	De Castro et al. (2013)
P5993	*E. coli* DH10B Δ*wecA::FRT*	Liu et al. (2017)
P6124	*E. coli* SΦ874 Δ*galE::FRT*	This study
SΦ874	*E. coli lacZ4503 trp‐355 upp‐12 relA rpsL150* Δ*(sbc‐rfb)86*	Neuhard and Thomassen (1976)

**Table 2:** Plasmids used in this study

Plasmid	Description^a^	Source/Reference
pPR981	Low‐copy cosmid containing the entire M85 O:2a O‐antigen gene cluster. Kan^R^, Strep^R^, Spec^R^	Kessler et al. (1991)
pPR1213	*Sal*I fragment of pPR981 containing the 3΄ half of the O:2a O‐antigen gene cluster cloned into pUC18. Amp^R^	Kessler et al. (1991)
pPR1214	*Sal*I fragment of pPR981 containing the 5΄ half of the O:2a O‐antigen gene cluster cloned into pUC18. Amp^R^	Kessler et al. (1991)
pPR2182	The M85 *wbyA* gene cloned into pTrc99A. Amp^R^	This study
pPR2216	The *S. enterica* LT2 *abe* gene cloned into pTrc99A. Amp^R^	Hong and Reeves (2015)
pPR2252	Yeast‐*E. coli* shuttle vector with hooks from *E. coli* K‐12 *galF* and *gnd* and *Y. pseudotuberculosis* O:1b *ddhD* and *wzz* for recombinational cloning of O‐antigen gene clusters. Amp^R^, Cml^R^ (*E. coli*); Cyh^S^ (yeast)	Liu et al. (2017)
pPR2272	The M85 O:2a O‐antigen gene cluster cloned into pPR2252 (O:2a WT). Cml^R^	This study
pPR2283	The M451 *wzx*_*3*_ gene cloned into pWQ552. Amp^R^	This study
pPR2284	The M2575 *wzx*_*7*_ gene cloned into pWQ552. Amp^R^	This study
pPR2285	The M2577 *wzx*_*8*_ gene cloned into pWQ552. Amp^R^	This study
pPR2288	The M85 *wzx*_*2*_ gene cloned into pWQ552. Amp^R^	This study
pPR2289	The M460 *wzx*_*4*_ gene cloned into pWQ552. Amp^R^	This study
pPR2290	The M462 *wzx*_*5*_ gene cloned into pWQ552. Amp^R^	This study
pPR2293	The M444 *wzx*_*1*_ gene cloned into pWQ552. Amp^R^	This study
pPR2296	pPR2272 with the *wzx*_*2*_ gene replaced by an *rpsL‐kan* cassette (O:2a Δ*wzx*). Cml^R^, Kan^R^	This study
pPR2297	pPR2272 with the abe‐tyv* region replaced by an *rpsL‐kan* cassette (O:2a Δ*abe*). Cml^R^, Kan^R^	This study
pPR2299	pPR2272 with the *wzx*_*2*_ gene replaced by the M444 *wzx*_*1*_ gene (O:2a‐*wzx*_*1*_). Cml^R^	This study
pPR2300	pPR2272 with the abe‐tyv* region replaced by the M454 *prt‐tyv* region (O:4b WT). Cml^R^	This study
pPR2303	pPR2272 with the *wzx*_*2*_ gene replaced by the M451 *wzx*_*3*_ gene (O:2a‐*wzx*_*3*_). Cml^R^	This study
pPR2304	pPR2272 with the *wzx*_*2*_ gene replaced by the M460 *wzx*_*4*_ gene (O:2a‐*wzx*_*4*_). Cml^R^	This study
pPR2305	pPR2272 with the *wzx*_*2*_ gene replaced by the M462 *wzx*_*5*_ gene (O:2a‐*wzx*_*5*_). Cml^R^	This study
pPR2306	pPR2272 with the *wzx*_*2*_ gene replaced by the M2575 *wzx*_*7*_ gene (O:2a‐*wzx*_*7*_). Cml^R^	This study
pPR2307	pPR2272 with the *wzx*_*2*_ gene replaced by the M2577 *wzx*_*8*_ gene (O:2a‐*wzx*_*8*_). Cml^R^	This study
pPR2310	pPR2300 with the *wzx*_*2*_ gene replaced by an *rpsL‐kan* cassette (O:4b Δ*wzx*). Cml^R^, Kan^R^	This study
pPR2313	pPR2300 with the *wzx*_*2*_ gene replaced by the M444 *wzx*_*1*_ gene (O:4b‐*wzx*_*1*_). Cml^R^	This study
pPR2314	pPR2272 with the abe‐tyv* region and *wbyA* gene replaced by the M444 *prt‐wbyH* region and *wbyM* gene, respectively (O:1a‐*wzx*_*2*_). Cml^R^	This study
pPR2315	pPR2272 with the abe‐tyv* and *wzx*_*2*_ *‐wbyA* regions replaced by the M444 *prt‐wbyH* and *wzx*_*1*_ *‐wbyM* regions, respectively (O:1a WT). Cml^R^	This study
pPR2320	The M444 *prt‐wbyH* region cloned into pTrc99A. Amp^R^	This study
pPR2321	The M454 *prt‐tyv* region cloned into pTrc99A. Amp^R^	This study
pPR2322	pPR2300 with the *tyv* gene replaced by an *rpsL‐kan* cassette (O:4b Δ*tyv*). Cml^R^, Kan^R^	This study
pPR2323	pPR2313 with the *tyv* gene replaced by an *rpsL‐kan* cassette (O:4b‐*wzx*_*1*_ Δ*tyv*). Cml^R^, Kan^R^	This study
pPR2326	pPR2315 with the *prt‐wbyH* region replaced by an *rpsL‐kan* cassette (O:1a Δ*prt‐wbyH*). Cml^R^, Kan^R^	This study
pPR2327	pPR2315 with the *wzx*_*1*_ gene replaced by an *rpsL‐kan* cassette (O:1a Δ*wzx*). Cml^R^, Kan^R^	This study
pTrc99A	Expression vector with pBR322 *ori*, IPTG‐inducible promoter (P_*trc*_), Amp^R^	Amann, Ochs, and Abel (1988)
pWQ552	Expression vector with p15A *ori*, tetracycline‐inducible promoter (P_*tet*_), Amp^R^	Willis and Whitfield (2013)

a Underlined descriptors indicate the plasmid names as shown in the main text. Abbreviations: ampicillin (Amp^R^), chloramphenicol (Cml^R^), kanamycin (Kan^R^), spectinomycin (Spec^R^), streptomycin (Strep^R^) resistance; cycloheximide sensitivity (Cyh^S^).

### 2.2 PCR and oligonucleotides

All PCR products were amplified with Q5 HF DNA Polymerase (New England Biolabs; NEB) and purified with the Isolate II PCR and Gel Kit (Bioline) as per the manufacturers’ instructions. All oligonucleotides used in this study are described in Supporting Information Table S1.

### 2.3 Strain manipulation

The SΦ874 Δ*galE* strain (P6124) was generated, using the lambda‐RED recombinase system (Datsenko & Wanner, 2000), with the *galE* gene replaced by a kanamycin‐resistance cassette, which was subsequently excised by the FLP recombinase. Sanger sequencing (Australian Genome Research Facility; AGRF) was performed across the insertion junctions to confirm the gene knockout.

### 2.4 Single‐gene cloning and bacterial transformation

PCR products and vectors were double‐digested with the appropriate restriction enzymes (NEB high‐fidelity range), and digested vectors were dephosphorylated with recombinant shrimp alkaline phosphatase (NEB) under the standard conditions described by the manufacturer. Digested DNA was gel‐purified with the Wizard SV Gel and PCR Clean‐up Kit (Promega), and ligations were performed overnight at 16°C, using T4 DNA ligase (NEB) as per the manufacturer's instructions. Ligated DNA was transformed into CaCl_2_‐treated *E. coli* DH5α (Lederberg & Cohen, 1974), with putative clones isolated with the Purelink Quick Plasmid Miniprep Kit (Invitrogen). Sanger sequencing (AGRF) was used for confirmation of clones. Plasmids were transformed into the relevant *E. coli* strains by electroporation as described previously by Hong and Reeves (2014).

### 2.5 Cloning and manipulation of O‐antigen gene clusters

The “Operon Assembly Protocol”, described previously by Liu, Kenyon, Lee, and Reeves (2017), was used for assembly of the O:2a O‐antigen gene cluster onto the yeast‐*E. coli* Operon Assembly Vector (OAV; pPR2252) as a single‐copy clone. The OAV contains the *Y. pseudotuberculosis* O:2a *ddhD* and *wzz* “hook” sequences flanked by the *E. coli* K‐12 *galF*, JUMPStart and *gnd* sequences (Supporting Information Figure S3), meaning that expression of the cloned O‐antigen gene clusters are under the control of the K‐12 JUMPStart region (Bailey, Hughes, & Koronakis, 1997). Three DNA fragments were used to assemble the O:2a WT clone (Supporting Information Figure S3): the *Sal*I fragments from the O:2a subclones pPR1213 and pPR1214, which contain the 3΄ and 5΄ halves of the O:2a gene cluster, respectively, and an overlapping ~4.4 kb *Hin*dIII fragment from pPR981, a multicopy cosmid clone of the O:2a O‐antigen gene cluster (Kessler, Brown, Romana, & Reeves, 1991). These fragments and the *Sma*I‐digested OAV were transformed into *S. cerevisiae* CRY1‐2, and recombinant clones were subsequently isolated and recovered in *E. coli* strain P5993 as per the standard OAP methodology. Functional testing of the O:2a WT clone (pPR2272) showed that it was producing a similar LPS profile to pPR981 when expressed in SΦ874 (Supporting Information Figure S4).

All subsequent O‐antigen gene cluster clones were generated by targeted gene knockouts and/or replacements using the modified version of the lambda‐RED recombinase system described by Hong, Cunneen, and Reeves (2012), where genes were replaced by an *rpsL‐kan* cassette under kanamycin selection, which was subsequently replaced by the gene of interest under high‐level streptomycin selection. All gene replacements were performed in P5993, which has an *rpsL* mutation conferring high‐level streptomycin resistance. Sanger sequencing (AGRF) was performed across the entire length of replacement genes or cassettes.

### 2.6 LPS extraction and visualization

Bacterial strains were subcultured 1:100 from overnight cultures and incubated, using the appropriate growth medium, selection and temperature, with shaking at 200 rpm. Overexpression of single‐gene clones was induced at OD_600_ ~ 0.3 with tetracycline (50 ng/ml) for pWQ552‐based clones or IPTG (1 mM) for pTrc99A‐based clones (Table 2). D‐Gal (0.2% (w/v)) was added at OD_600_ ~ 0.5 to activate O‐antigen production in Δ*galE* strains, and cells were harvested at OD_600_ ~ 0.7.

LPS extractions were performed as described by McGrath and Osborn (1991), but with the modifications described by Liu et al. (2017). Samples were separated by tricine‐SDS‐PAGE on 13% (v/v) polyacrylamide gels, with loadings standardized relative to the densities of the lipid A‐core bands of each sample. LPS was visualized by silver staining (Brown, Romana, & Reeves, 1991) or transferred to a Hybond‐C Extra nitrocellulose membrane (Amersham Biosciences) for immunoblot analysis (Howland, 1996). Membranes were incubated with polyclonal anti‐*Salmonella* O2, O4 or O9 antisera (Statens Serum Institut), followed by goat‐anti‐rabbit‐IgG‐HRP‐conjugated antibody (Pierce). Chromogenic detection was performed with 0.3% (w/v) 4‐chloro‐1‐napthol, 0.075% (v/v) H_2_O_2_ and 10% (v/v) methanol in TBS‐Tween solution. Silver‐stained gels and Western blots were imaged in a ChemiDoc MP Imaging System (Bio‐Rad).

## 3 RESULTS

### 3.1 Cloning and expression of wild‐type *Y. pseudotuberculosis* O‐antigen gene clusters

Expression of a given O‐antigen gene cluster can often vary dramatically across different strains, due to the actions of unidentified or poorly understood regulatory factors (Lerouge & Vanderleyden, 2002). To facilitate meaningful comparisons between the *Y. pseudotuberculosis* O:1a, O:2a and O:4b O‐antigens and their Wzx flippases, we cloned the respective O‐antigen gene clusters and expressed them in a common genetic background. The *E. coli* strain SΦ874, which has a deletion of its entire O‐antigen gene cluster, was selected as the tester strain. An SΦ874 Δ*galE* strain (P6124) was constructed to allow control of O‐antigen production by the presence or absence of exogenous Gal and avoid potential lethality, as the shared O‐unit main‐chain has Gal as the second sugar, and attachment of the second sugar is the first committed step in O‐antigen biosynthesis.

**Figure 2:**
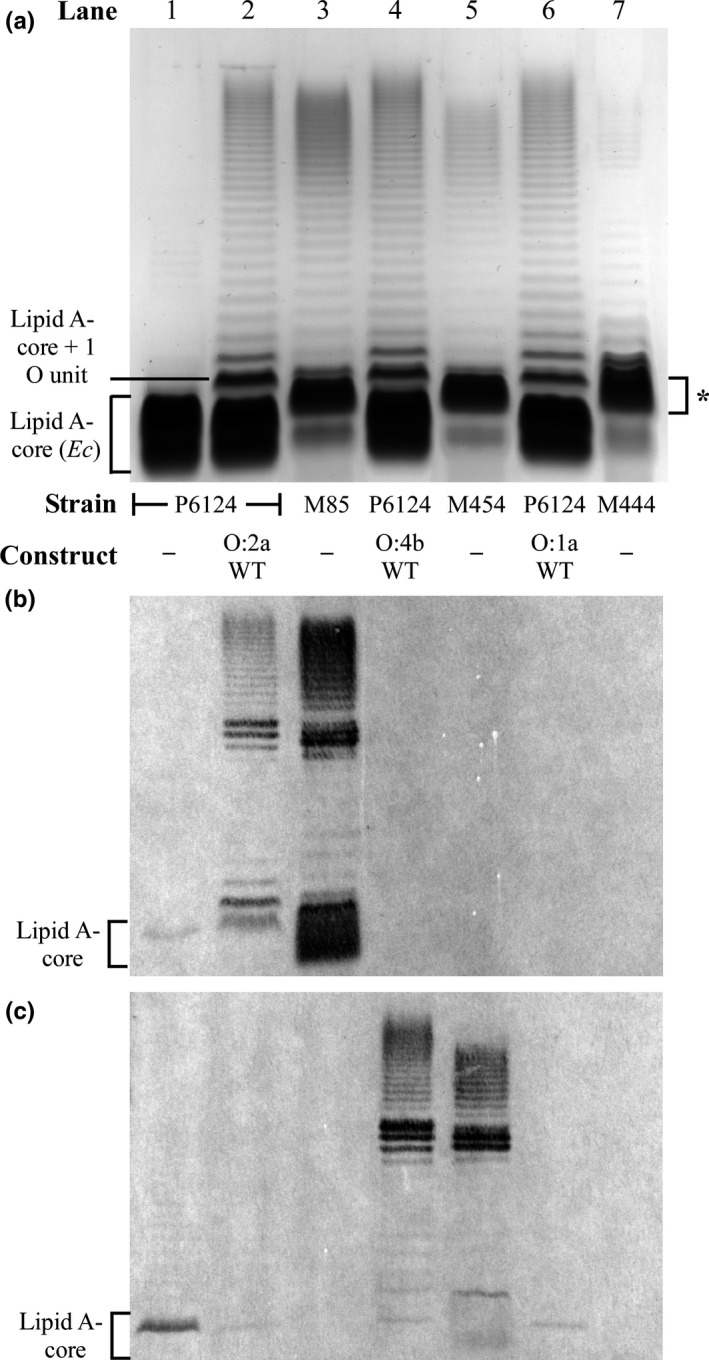
LPS profiles of the WT O:1a, O:2a and O:4b O‐antigen gene cluster clones. LPS samples were extracted from the indicated *Y. pseudotuberculosis* strains or P6124 harboring the indicated O‐antigen gene cluster constructs. The LPS was separated on a 13% (v/v) polyacrylamide gel by tricine‐SDS‐PAGE, followed by detection via silver staining (a) or transfer to a nitrocellulose membrane for immunoblotting with the *S. enterica* O4 (b) or O9 (c) antisera. The band corresponding to *E. coli* LPS without O antigen (lipid A‐core) is indicated to the left of the gel, while the equivalent band from *Y. pseudotuberculosis* is indicated by the asterisk on the right. The apparent location of these lipid A‐core only bands, which reacted nonspecifically with the antisera, are shown to the left of each membrane. The band corresponding to *E. coli* LPS with a single O unit (lipid A‐core + 1 O unit) is also shown on the gel

An O:2a WT clone (pPR2272) was constructed, using the Operon Assembly Protocol (Liu et al., 2017), resulting in the gene cluster being located on a plasmid with a mini‐F replicon, for which the copy number approximates the number of chromosomes per cell (Kline, 1985). The gene cluster is positioned downstream of the K‐12 O‐antigen regulatory system, including the JUMPStart sequence (Bailey et al., 1997), so expression is expected to be similar to normal chromosomal levels. An artificial O:4b WT clone (pPR2300) was then constructed by replacing the O:2a WT *abe* and degraded *tyv* genes (abe‐tyv*) with the O:4b *prt‐tyv* region (Figure 1a), while an artificial O:1a WT clone (pPR2315) was similarly constructed by replacing the O:2a WT abe‐tyv* and *wzx*_*2*_ *‐wbyA* regions with the O:1a *prt‐wbyH* and *wzx*_*1*_ *‐wbyM* regions (Figure 1a), respectively. As a result, the shared regions are identical across the three gene cluster clones. All three constructs were expressed in P6124, where their LPS profiles were found to be indistinguishable (Figure 2a, lanes 2, 4, 6), in stark contrast to the varied LPS profiles of the parental *Y. pseudotuberculosis* strains (Figure 2a, lanes 3, 5, 7), reinforcing the benefits of reconstructing the gene clusters and comparing them in the same strain. The identical O:2a and O:4b LPS profiles (Figure 2a, lanes 2, 4) also confirms that the O:2a Wzx_2_ (99% similar to the O:4b Wzx_2_) is equally efficient at flipping O:2a and O:4b O units. It is important to note that LPS from P6124 and the *Y. pseudotuberculosis* strains migrate at different rates due to differences in the composition of the LPS core oligosaccharides (Amor et al., 2000; Knirel & Anisimov, 2012).

Immunoblotting was performed to confirm that the O:1a, O:2a and O:4b WT constructs were producing O antigen with the expected DDH structures. As expected, the Abe*p*‐specific *S. enterica* O4 antiserum reacted with the P6124 +  O:2a WT and M85 O:2a LPS (Figure 2b, lanes 2–3), while the Tyv*p*‐specific *S. enterica* O9 antiserum reacted with the P6124 +  O:4b WT and M454 O:4b LPS (Figure 2c, lanes 4–5), confirming that both constructs are producing the expected O‐antigen structures. A weak nonspecific reaction was also visible with both antisera against the lipid A‐core LPS of P6124 (Figure 2b–c, lane 1). While there is no commercially available antiserum for detection of Par*f* residues, several indirect lines of evidence indicate that the O:1a WT construct is producing O:1a O antigen. The migration rate for P6124 +  O:1a WT LPS is identical to its O:2a and O:4b counterparts (Figure 2a, lanes 2, 4, 6), suggesting the presence of a DDH residue. This residue is clearly not Abe*p* or Tyv*p*, as neither P6124 +  O:1a WT nor M444 O:1a LPS reacted with the O4 or O9 antisera (Figure 2b–c, lanes 6–7). Finally, these LPS samples also failed to react specifically with a paratose (Par*p*)‐specific *S. enterica* O2 antiserum (data not shown), leaving Par*f* as by far the most likely residue.

It is noteworthy that the O4 and O9 antisera both exhibited a stronger reaction with the parental *Y. pseudotuberculosis* O:2a and O:4b LPS than with the equivalent *E. coli* LPS (Figure 2b, lanes 2–3; Figure 2c, lanes 4–5), which did not reflect the different levels of O antigen as observed by silver staining (Figure 2a, lanes 2–5). A possible explanation for this difference may be that the bacteriophage‐encoded O‐antigen glucosylation system in *E. coli* K‐12 (Mann, Ovchinnikova, King, & Whitfield, 2015) is almost certainly adding a glucose (Glc) residue onto the GlcNAc of the O antigens. The presence of these Glc residues may be interfering with the interaction of the antisera with their target epitopes, resulting in a weakened immunoblot signal. However, this was not problematic, as the immunoblots were only intended to qualitatively identify the presence or absence of Abe*p* and Tyv*p* residues, which was successfully achieved. Importantly, glucosylation of O antigens by this system occurs in the periplasm after Wzx translocation (Allison & Verma, 2000), and thus has no effect on our ability to interpret Wzx translocation efficiency.

### 3.2 Wzx_1_ and Wzx_2_ are both inefficient at flipping O units without a DDH residue

Removal of the terminal DDH residue from several *S. enterica* Gal‐initiated O antigens results in poor Wzx translocation (Hong et al., 2012; Liu et al., 2015, 2017). To assess this situation for the *Y. pseudotuberculosis* Wzx_1_ and Wzx_2_, we made O:2a Δ*abe* (pPR2297) and O:1a Δ*prt‐wbyH* (pPR2326) constructs that both produce the same O unit main‐chain without a DDH residue, but express *wzx*_*2*_ or *wzx*_*1*_, respectively.

**Figure 3:**
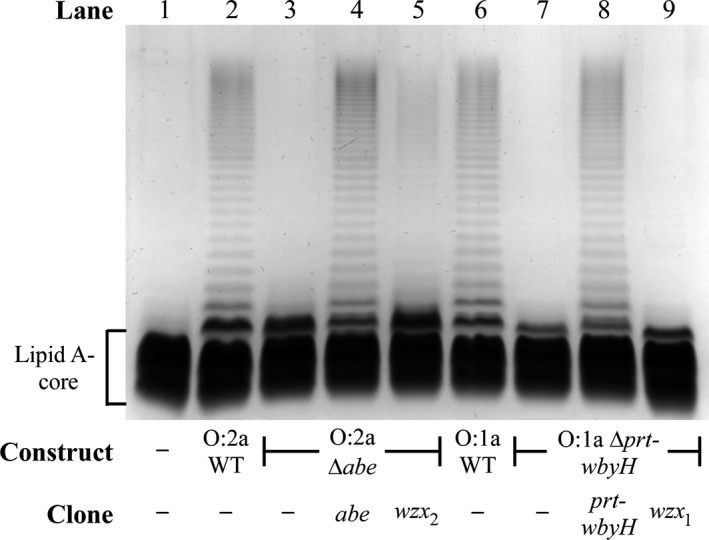
LPS profiles of the *Y. pseudotuberculosis* O‐antigen gene cluster clones expressing O antigen without a DDH residue. LPS samples were extracted from P6124 harboring the indicated O‐antigen gene cluster constructs and single‐gene clones, and separated on a 13% (v/v) polyacrylamide gel by tricine‐SDS‐PAGE, followed by detection via silver staining. The location of LPS without O antigen (lipid A‐core) is indicated

These two constructs were transformed into P6124 and their LPS profiles analyzed by SDS‐PAGE. O‐antigen production from the O:2a Δ*abe* construct (Figure 3, lane 3) was severely reduced relative to the O:2a WT construct (Figure 3, lane 2), and the LPS also migrated faster than WT, as expected for truncated O units. The O:1a Δ*prt‐wbyH* construct similarly produced faster‐migrating LPS (Figure 3, lane 7), but with even lower O‐antigen levels than the O:2a Δ*abe* construct (Figure 3, lane 3). Complementation of these two constructs with *abe* (pPR2216) or *prt‐wbyH* clones (pPR2320) fully restored WT O‐antigen levels and LPS migration rates (Figure 3, lanes 4, 8), confirming that the observed effects were not due to mutational polarity effects.

Wzx overexpression has previously been shown to improve the otherwise inefficient translocation of truncated O units (Hong & Reeves, 2014; Liu et al., 2015), so we overexpressed tetracycline‐inducible *wzx*_*2*_ (pPR2288) and *wzx*_*1*_ (pPR2293) clones alongside the O:2a Δ*abe* and O:1a Δ*prt‐wbyH* constructs, respectively. In both instances, O‐antigen levels increased (Figure 3, lanes 5, 9), with more detectable single‐O‐unit LPS for both constructs, and a small amount of long‐chain O antigen for the O:2a Δ*abe* construct, but both remained well below WT levels (Figure 3, lanes 2, 6). Although this did confirm that both Wzx are inefficient at translocating the truncated O unit and that Wzx_2_ is more efficient than Wzx_1_, we cannot exclude the possibility that Wzy is also inefficient at polymerizing these truncated O units, making it impossible to determine the extent of Wzx inefficiency.

### 3.3 Wzx_1_ and Wzx_2_ show varied efficiency for flipping O‐units with alternative DDH residues

We next constructed O‐antigen gene cluster clones containing the alternative *wzx* (O:2a‐*wzx*_*1*_ (pPR2299), O:4b‐*wzx*_*1*_ (pPR2313) and O:1a‐*wzx*_*2*_ (pPR2314)) to compare the translocation efficiencies of Wzx_1_ and Wzx_2_ with the alternative DDH structure(s) on a common O‐unit main‐chain. Equivalent *wzx* knockout clones (O:2a Δ*wzx* (pPR2296), O:4b Δ*wzx* (pPR2310) and O:1a Δ*wzx* (pPR2327)) were also constructed to act as comparison controls.

**Figure 4:**
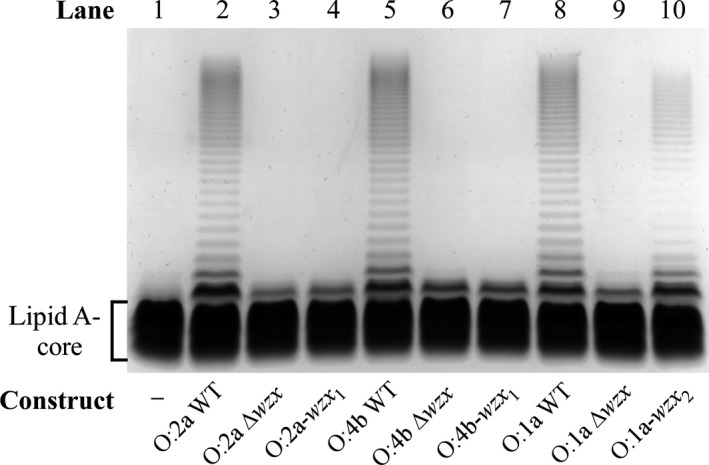
LPS profiles of O:1a, O:2a and O:4b O‐antigen gene cluster clones with the alternative *wzx* genes. LPS samples were extracted from P6124 harboring the indicated O‐antigen gene cluster constructs and separated on a 13% (v/v) polyacrylamide gel by tricine‐SDS‐PAGE, followed by detection via silver staining. The location of LPS without O antigen (lipid A‐core) is indicated

These constructs were transformed into P6124 and their LPS profiles analyzed by SDS‐PAGE. Comparison of the O:1a, O:2a and O:4b Δ*wzx* constructs with their WT counterparts revealed near‐complete loss of O‐antigen production (Figure 4, lanes 2–3, 5–6, 8–9), with the exception of a small amount of single‐O‐unit LPS that likely reflects background translocation by the *E. coli* colanic acid or enterobacterial common antigen flippases. The O:2a‐*wzx*_*1*_ and O:4b‐*wzx*_*1*_ constructs produced O‐antigen levels that were indistinguishable from their Δ*wzx* counterparts (Figure 4, lanes 3–4, 6–7), demonstrating that Wzx_1_ is effectively unable to flip the O:2a and O:4b O units. The O:1a‐*wzx*_*2*_ construct also had notably reduced O‐antigen levels relative to its WT counterpart (Figure 4, lanes 8, 10), demonstrating that while Wzx_2_ is clearly inefficient at flipping O:1a O units, this inefficiency is far less dramatic than observed for Wzx_1_ with the O:2a and O:4b O units (Figure 4, lanes 4, 7). When complemented with overexpressed cognate *wzx* clones, all *wzx* deletion and replacement constructs produced WT O‐antigen levels (Supporting Information Figure S1), confirming that the observed reductions in O antigen were exclusively due to the change in *wzx*. Overall, these results clearly demonstrate that both Wzx can distinguish between different terminal DDH residues with only minor differences in structure and/or linkage.

**Figure 5:**
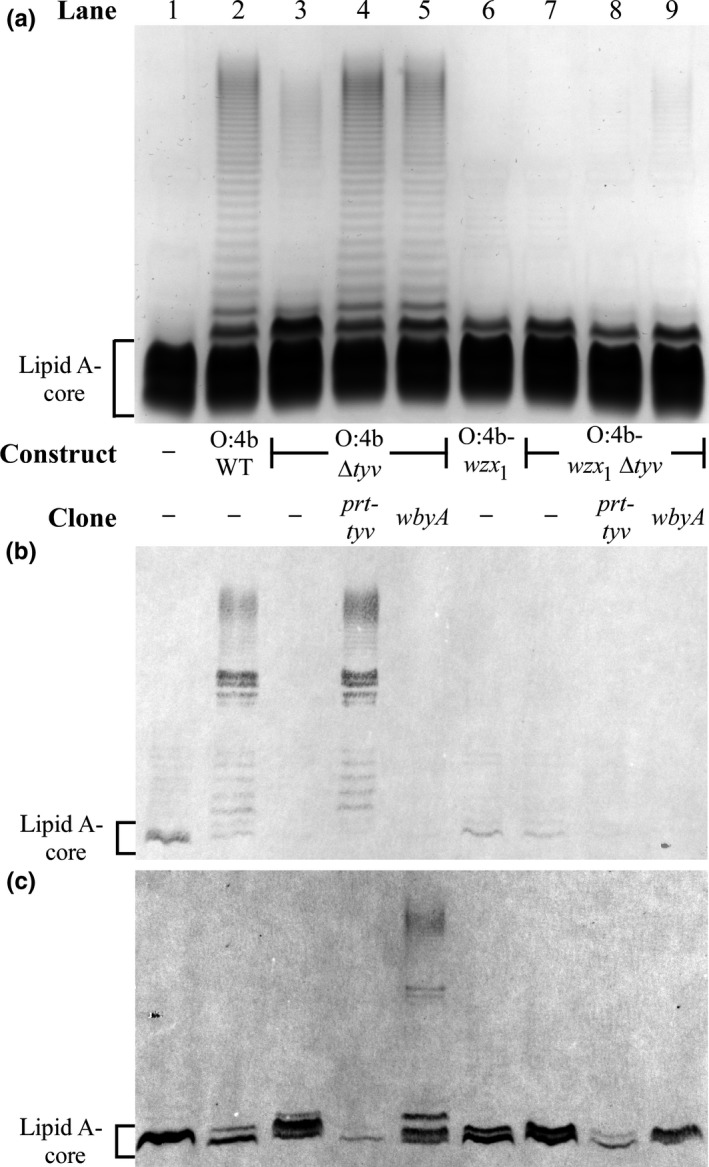
LPS profiles of *Y. pseudotuberculosis* O‐antigen gene cluster clones expressing O units with Par*p*. LPS samples were extracted from P6124 harboring the indicated O‐antigen gene cluster constructs and single‐gene clones, and separated on a 13% (v/v) polyacrylamide gel by tricine‐SDS‐PAGE, followed by detection via silver staining (a) or transfer to a nitrocellulose membrane for immunoblotting with the *S. enterica* O9 (b) or O2 (c) antisera. The location of LPS without O antigen (lipid A‐core), which reacted nonspecifically with the antisera, is indicated to the left of the gel and membranes

Our analysis was further expanded to explore whether Wzx_1_ or Wzx_2_ could efficiently translocate the equivalent O unit with an α(1,3)‐linked terminal Par*p* residue. This involved synthesis of a structure not found in nature, so the possibility of the terminal sugar GT or Wzy being inefficient at processing the O unit also had to be considered. Par*p* is the precursor sugar for Tyv*p*, so deletion of the *tyv* gene results in the synthesis of Par*p* exclusively, hence O:4b Δ*tyv* (pPR2322) and O:4b‐*wzx*_*1*_ Δ*tyv* (pPR2323) clones were constructed. These two constructs were expressed in P6124 and their LPS profiles analyzed by SDS‐PAGE. The O‐antigen levels from the *wzx*_*2*_‐expressing O:4b Δ*tyv* construct (Figure 5a, lane 3) were significantly reduced relative to the O:4b WT construct (Figure 5a, lane 2), although substantial short‐chain and some long‐chain O antigen was still detectable. The low‐level O‐antigen produced by the O:4b‐*wzx*_*1*_ Δ*tyv* construct (Figure 5a, lane 7) was similar to the O:4b‐*wzx*_*1*_ construct (Figure 5a, lane 6). Introduction of a *prt‐tyv* clone (pPR2321) reverted O‐antigen levels from both Δ*tyv* constructs to that of their parental constructs (Figure 5a, lanes 4, 8).

To determine if inefficient attachment of Par*p* by WbyA was a factor affecting O‐antigen production for the Δ*tyv* constructs, an IPTG‐inducible *wbyA* clone (pPR2182) was introduced and overexpressed. For the *wzx*_*2*_‐expressing O:4b Δ*tyv* construct, this dramatically increased O‐antigen production (Figure 5a, lane 5) to levels that were indistinguishable from its WT counterpart (Figure 5a, lane 2). This indicates that WbyA is inefficient at Par*p* attachment, but once this inefficiency is overcome, Wzx_2_ can flip the available Par*p*‐containing O units with similar efficiency to its cognate O:2a and O:4b O units. In contrast, for the O:4b‐*wzx*_*1*_ Δ*tyv* construct, *wbyA* overexpression only minimally increased O‐antigen levels, including a very small amount of long‐chain O antigen (Figure 5a, lane 9). Hence it appears that Wzx_1_ is much less efficient than Wzx_2_ at translocating the Par*p*‐containing O units, but is more efficient than with the equivalent Abe*p*‐ and Tyv*p*‐containing O units (Figure 4, lanes 4, 7).

Immunoblotting was used to confirm the predicted DDH residues on the O antigen from these constructs. O:4b WT and O:4b Δ*tyv* + *prt‐tyv* LPS were both shown to contain Tyv*p* residues based on their strong reaction with the *S. enterica* O9 antiserum (Figure 5b, lanes 2, 4). Low‐level O9 background reactivity was also observed with the lipid A‐core band of several LPS samples (Figure 5b, lanes 1, 6, 7). The only specific (albeit weak) reaction detected with the Par*p*‐specific *S. enterica* O2 antiserum was with long‐chain LPS from O:4b Δ*tyv* + *wbyA* (Figure 5c, lane 5), which confirmed that the O antigen produced by the O:4b Δ*tyv* construct does contain Par*p*, and that Wzx_2_ can efficiently flip these O units following WbyA overproduction. The remaining LPS samples exhibited varying degrees of nonspecific lipid A‐core cross‐reactivity with the O2 antiserum, which in combination with the very low levels of O antigen produced by the O:4b‐*wzx*_*1*_ Δ*tyv* construct, prevented the positive identification of Par*p*‐containing O units from this construct (Figure 5c, lanes 7, 9). However, given that the same deletion in the O:4b Δ*tyv* construct clearly resulted in the production of Par*p*‐containing O units (Figure 5c, lane 5), it is highly likely that the same is occurring for the O:4b‐*wzx*_*1*_ Δ*tyv* construct.

### 3.4 Several unrelated Wzx can efficiently translocate the O:2a O unit

We next attempted to compare the abilities of other *Y. pseudotuberculosis* Wzx flippases with more structurally divergent cognate O units to flip the O:2a O unit. The flippases selected were the O:3 Wzx_3_, O:6 Wzx_4_, O:7 Wzx_5_, O:10 Wzx_7_ and O:12 Wzx_8_, whose cognate O units all contain a DDH residue (Fig. S2). The *wzx*_*3*_ and *wzx*_*8*_ genes represent the other two *wzx* types found within the 14‐related *Y. pseudotuberculosis* O‐antigen gene clusters, whereas *wzx*_*4*_ and *wzx*_*5*_ are from unrelated gene clusters (Kenyon et al., 2017).

**Figure 6:**
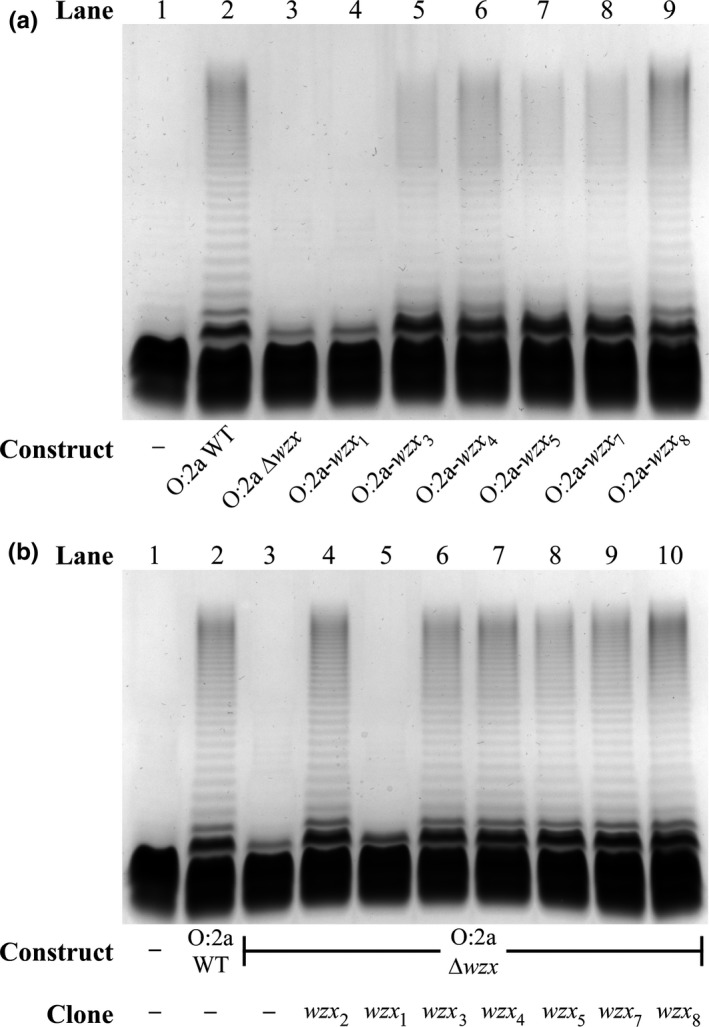
LPS profiles of the O:2a O‐antigen gene cluster clone containing alternative *wzx* genes. LPS samples were extracted from P6124 harboring the O:2a construct with *wzx* replaced (a) or the O:2a Δ*wzx* construct alongside the indicated overexpressed *wzx* clones (b). Samples were separated on a 13% (v/v) polyacrylamide gel by tricine‐SDS‐PAGE, followed by detection via silver staining.

O:2a clones with the *wzx*_*2*_ gene replaced by the alternative *wzx* (O:2a‐*wzx*_*3*_ (pPR2303), O:2a‐*wzx*_*4*_ (pPR2304), O:2a‐*wzx*_*5*_ (pPR2305), O:2a‐*wzx*_*7*_ (pPR2306) and O:2a‐*wzx*_*8*_ (pPR2307)) were constructed and expressed in P6124, and their LPS profiles analyzed by SDS‐PAGE. In contrast to the O:2a‐*wzx*_*1*_ construct (Figure 6a, lane 4), which produced O‐antigen levels indistinguishable from the O:2a Δ*wzx* construct (Figure 6a, lane 3), all other *wzx* replacement constructs produced at least some detectable long‐chain O antigen (Figure 6a, lanes 5–9). The O‐antigen levels from the O:2a‐*wzx*_*8*_ construct (Figure 6a, lane 9) were indistinguishable from the O:2a WT construct (Figure 6a, lane 2), while the O:2a‐*wzx*_*4*_ construct (Figure 6a, lane 6) produced O‐antigen levels only slightly below WT. The O:2a‐*wzx*_*3*_, O:2a‐*wzx*_*5*_ and O:2a‐*wzx*_*7*_ constructs (Figure 6a, lanes 5, 7, 8) had reduced O‐antigen levels not dissimilar from those observed previously for the O:1a‐*wzx*_*2*_ construct (Figure 4, lane 10).

Overexpression of tetracycline‐inducible clones of each alternative *wzx* (*wzx*_*1*_, pPR2293; *wzx*_*3*_, pPR2283; *wzx*_*4*_, pPR2289; *wzx*_*5*_, pPR2290; *wzx*_*7*_, pPR2284; *wzx*_*8*_, pPR2285) restored O‐antigen production from the O:2a Δ*wzx* construct (Figure 6b, lanes 6–10) to O:2a WT levels (Figure 6b, lane 2), except for *wzx*_*1*_ (Figure 6b, lane 5), which was effectively indistinguishable from the O:2a Δ*wzx* construct alone (Figure 6b, lane 3). This *wzx*_*1*_ clone is known to be functional, as it can fully restore O‐antigen production from the O:1a Δ*wzx* construct (Supporting Information Figure S1). It is apparent that Wzx_1_ is extremely inefficient at translocating the O:2a O unit, being less efficient than other Wzx flippases with cognate O units that are more structurally‐divergent from the O:2a O unit.

## 4 DISCUSSION

### 4.1 Wzx flippases can exhibit both strict and relaxed substrate preferences

Our understanding of the nature of Wzx substrate specificity has been continuously evolving over time, ranging from Wzx sequence diversity being seen as related to O‐unit diversity, through relaxed Wzx substrate specificity being driven only by the identity of the first O‐unit residue (Feldman et al., 1999; Marolda et al., 2004), to Wzx specificity extending along the length of its cognate O‐unit structure (Hong & Reeves, 2014; Hong et al., 2012). By undertaking a detailed analysis of the translocation efficiencies of *Y. pseudotuberculosis* Wzx with a series of closely related O‐unit structures (data summarized in Table 3), we have discovered that Wzx substrate preference can be more complex than either previously‐suggested model.

**Table 3:** Comparison of Wzx repeat‐unit translocation efficiencies

Repeat unit name^a^	Repeat unit structure^b^	Wzx (efficiency)^c^,^d^	Reference
*Yp* O:2a	Abe*p*‐(α1→3)‐D‐6dManHep*p*‐(β1→4)‐D‐Gal*p*‐(α1→3)‐D‐Glc*p*NAc→	*Yp* Wzx_1_ (˗) ***Yp*** **Wzx**_**2**_ (+++) *Yp* Wzx_3_ (+) *Yp* Wzx_4_ (++) *Yp* Wzx_5_ (+) *Yp* Wzx_7_ (+) *Yp* Wzx_8_ (+++)	This study
*Yp* O:4b	Tyv*p*‐(α1→3)‐D‐6dManHep*p*‐(β1→4)‐D‐Gal*p*‐(α1→3)‐D‐Glc*p*NAc→	*Yp* Wzx_1_ (˗) ***Yp*** **Wzx**_**2**_ (+++)	
*Yp* O:1a	Par*f*‐(β1→3)‐D‐6dManHep*p*‐(β1→4)‐D‐Gal*p*‐(α1→3)‐D‐Glc*p*NAc→	***Yp*** **Wzx**_**1**_ (+++) *Yp* Wzx_2_ (++)	
*Yp* O:4b‐Par*p*	Par*p*‐(α1→3)‐D‐6dManHep*p*‐(β1→4)‐D‐Gal*p*‐(α1→3)‐D‐Glc*p*NAc→	*Yp* Wzx_1_ (+) *Yp* Wzx_2_ (+++)	
*Yp* O:2a ΔAbe*p*	D‐6dManHep*p*‐(β1→4)‐D‐Gal*p*‐(α1→3)‐D‐Glc*p*NAc→	*Yp* Wzx_1_ (˗) *Yp* Wzx_2_ (+)	
*Se* gp B	Abe*p*‐(α1→3)‐D‐Man*p*‐(α1→4)‐L‐Rha*p*‐(α1→3)‐D‐Gal*p*→	***Se*** **Wzx**_**B**_ (+++) *Se* Wzx_D_ (+++)	Hong et al. (2012)
*Se* gp D	Tyv*p*‐(α1→3)‐D‐Man*p*‐(α1→4)‐L‐Rha*p*‐(α1→3)‐D‐Gal*p*→	*Se* Wzx_B_ (+++) ***Se*** **Wzx**_**D**_ (+++) *Se* Wzx_E_ (+)	
*Se* gp E	D‐Man*p*‐(α1→4)‐L‐Rha*p*‐(α1→3)‐D‐Gal*p*→	*Se* Wzx_B_ (+)	
		*Se* Wzx_D_ (+)	
		***Se*** **Wzx**_**E**_ (+++)	
*Se* gp C2	Abe*p*‐(α1→3)‐L‐Rha*p*‐(α1→2)‐D‐Man*p*‐(α1→2)‐D‐Man*p*‐(α1→3)‐D‐Gal*p*→	***Se*** **Wzx**_**C**_ (+++)	Liu et al. (2015)
*Se* gp C2 ΔAbe*p*	L‐Rha*p*‐(α1→2)‐D‐Man*p*‐(α1→2)‐D‐Man*p*‐(α1→3)‐D‐Gal*p*→	*Se* Wzx_C_ (+)	
*Ec* O1A	D‐Man*p*NAc‐(β1→2)‐L‐Rha*p*‐(α1→3)‐L‐Rha*p*‐(α1→3)‐L‐Rha*p*‐(β1→4)‐D‐Glc*p*NAc→	***Ec*** **Wzx**_**O1A**_ (+++) *Ec* Wzx_O2_ (‐) *Se* Wzx_O42_ (+++)	Liu et al. (2017)
*Ec* O1A ΔMan*p*NAc	L‐Rha*p*‐(α1→3)‐L‐Rha*p*‐(α1→3)‐L‐Rha*p*‐(β1→4)‐D‐Glc*p*NAc→	***Ec*** **Wzx**_**O1A**_ (‐)	
*Ec* O16	OAc↓(2) D‐Gal*f*‐(β1→6)‐D‐Glc*p*‐(α1→3)‐L‐Rha*p*‐(α1→3)‐D‐Glc*p*NAc→	***Ec*** **Wzx**_**O16**_ (+++)	Hong and Reeves (2014)
		*Ec* Wzx_O7_ (++)	
		*Ec* Wzx_O111_ (+)	
		*Ec* Wzx_O157_ (‐)	
		*Sf* Wzx_2a_ (‐)	
		*Se* Wzx_B_ (‐)	
*Ec* O111	D‐Col*p* (α1↓3) D‐Glc*p*‐(α1→4)‐D‐Gal*p*‐(α1→3)‐D‐Glc*p*NAc→ (α1↑6) D‐Col*p*	***Ec*** **Wzx**_**O111**_ (+++) *Ec* Wzx_O16_ (++) *Ec* Wzx_O7_ (+) *Sf* Wzx_2a_ (‐)	
*Ea* amylovoran	Pyr‐(4,6)‐D‐Gal*p*‐(α1→4)‐D‐Glc*p*A‐(β1→4)‐D‐Gal*p*‐(α1→6)‐D‐Gal*p*‐(β1→3)‐D‐Gal*p*→	***Ea*** **AmsL1** (+++) *Ea* AmsL2 (˗)	Wang et al. (2012)
*Ps* stewartan	D‐Glc*p*‐(β1→6)‐D‐Gal*p*‐(α1→4)‐D‐Glc*p*A‐(β1→4)‐D‐Gal*p*‐(α1→6)‐D‐Glc*p*‐(β1→3)‐D‐Gal*p*→	*Ps* Wzx1 (˗) ***Ps*** **Wzx2** (+++)	

a The prefixes indicate the source species for each structure: *Erwinia amylovora* (*Ea*), *Escherichia coli* (*Ec*), *Pantoea stewartii* (*Ps*), *Salmonella enterica* (*Se*), *Shigella flexneri* (*Sf*) or *Yersinia pseudotuberculosis* (*Yp*).

b Repeat units are shown without their respective polymerization linkages, with residues displayed from last‐ to first‐sugar reading left‐to‐right, and the rightmost arrow denoting the undecaprenyl pyrophosphate linkage. All residue abbreviations are defined in‐text, except for D‐colitose (D‐Col*p*), D‐galactofuranose (D‐Gal*f*), D‐glucopyranuronic acid (D‐Glc*p*A), D‐mannose (D‐Man*p*), D‐*N*‐acetylmannosamine (D‐Man*p*NAc), *O*‐acetyl (OAc) and L‐rhamnose (L‐Rha*p*).

c The cognate Wzx for each repeat unit is **bolded**. Wzx translocation efficiencies for O units are ranked based on the amount of O antigen produced when Wzx is expressed at WT levels as follows: (˗) little to no detectable O antigen; (+) low‐level O‐antigen production with minimal long‐chain LPS; (++) moderate O‐antigen production with considerable long‐chain LPS that is below WT levels; (+++) O‐antigen production that is indistinguishable from WT levels.

d The Wzx translocation efficiencies for the exopolysaccharides amylovoran and stewartan are only ranked as (˗) or (+++), as their production levels were only described as being “detectable” or “undetectable”.

Elimination of the terminal DDH residue dramatically affected the O‐unit translocation efficiency of Wzx_1_ and Wzx_2_ in this study (Figure 3), which was similarly observed for the Wzx of the *S. enterica* Gal‐initiated O‐antigen set (Hong et al., 2012; Liu et al., 2015). More importantly, both these Wzx could also distinguish, to varying degrees, changes to the structure and/or linkage of the terminal DDH residue on a common O‐unit main‐chain. Wzx_1_ was severely deficient at flipping O units with α(1,3)‐linked Abe*p*, Tyv*p* or Par*p* residues, while Wzx_2_ was moderately deficient when translocating O units with a β(1,3)‐linked Par*f* residue (Table 3). Wang, Yang, and von Bodman (2012) previously proposed that two distinct Wzx pairs in *Pantoea stewartii* and *Erwinia amylovora* were required to translocate a pair of exopolysaccharides differing only by a Glc or pyruvate terminal side‐branch moiety, but did not directly confirm the role of each Wzx nor test the degree of specificity for each structure (Table 3). Hence, this study is the first to unambiguously demonstrate that Wzx substrate preference can be sensitive enough to be affected by minor alterations to the structure and/or linkage type of a single O‐unit residue.

**Figure 7:**
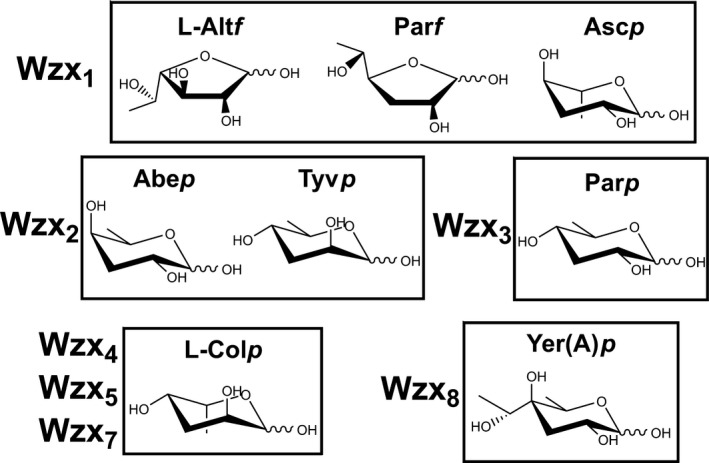
Structures of the DDH or DDH‐like terminal residues from *Y. pseudotuberculosis* O antigens. Residues grouped together into boxes are from O units flipped by the same Wzx, with the relevant Wzx indicated to the left of each box. Abbreviations: Abe*p*, abequose; L‐Alt*f*, L‐altrofuranose; Asc*p*, ascarylose; L‐Col*p*, L‐colitose; Par*f*, paratofuranose; Par*p*, paratose; Tyv*p*, tyvelose; Yer(A)*p*, yersiniose

In light of this high degree of specificity, it is surprising that Wzx_1_ and Wzx_2_ are both used for the translocation of several different O‐unit structures (Figure 1b). Wzx_1_ (94–95% similarity) translocates seven different O units with Par*f*, Alt*f* or Asc*p* terminal sugars, while Wzx_2_ (99% similarity) translocates five different O units with Abe*p* or Tyv*p* terminal sugars (Figure 7). Hence, both of these Wzx appear to be adapted to a defined set of terminal residues, rather than to entire O‐unit structures. However, other Wzx with more divergent cognate O‐unit structures were found to translocate the O:2a O unit with efficiencies ranging from moderate to indistinguishable from WT (Figure 6), indicating that the ability to translocate different O‐unit structures is common among the *Y. pseudotuberculosis* Wzx. Hong and Reeves (2014) also showed that several foreign Wzx were able to translocate the *E. coli* O16 and O111 O units with low‐to‐moderate efficiency (Table 3), indicating that partially‐relaxed Wzx substrate preference may be a widespread property.

### 4.2 A new model for Wzx substrate recognition

Wzx proteins belong to the polysaccharide transport (PST) family, one of 12 in the MOP (multidrug/oligosaccharide lipid/polysaccharide exporter) superfamily, and typically have 12 transmembrane segments (TMS). While there are currently no crystal structures available for Wzx or any other PST family member, the recently published structures of the related peptidoglycan flippase MurJ (Kuk, Mashalidis, & Lee, 2016) and *Campylobacter* oligosaccharide flippase PglK (Perez et al., 2015) have provided interesting insights. Both contain a central cavity that can be presented on either side of the IM, with transport proposed to occur by entry of the substrate into the cytoplasmic‐facing cavity conformation and exit via the periplasmic‐facing form, and it is likely that Wzx O‐unit translocation proceeds by a similar mechanism.

The combination of highly sensitive and relaxed substrate preference exhibited by Wzx_1_ and Wzx_2_ seems too complex to be explained by a simple lock‐and‐key model in which Wzx recognises the entire O‐unit structure. Rather, we propose “Structure‐Specific Triggering” as the mechanism, in which flipping is triggered by Wzx interacting with one or more specific structural elements on the complete O unit. We envisage specific sites within each Wzx that interact directly with the structural element(s) and trigger the translocation event. The full O‐unit structure would need to fit within the Wzx central cavity, and there may well be other critical requirements, such as a specific first sugar, but some changes to the composition and length of the O‐unit main‐chain have little effect on translocation efficiency for Wzx_1_ and Wzx_2_. Rapid translocation triggered by complete O units would mean that intermediates or unrelated foreign O units lacking the key structural element(s) are rarely flipped under normal circumstances. However, in cells unable to synthesize the complete O unit, other structural elements on the incomplete or foreign O units may occasionally interact with Wzx and trigger flipping at a reduced frequency. Overexpression of *wzx* can at least partially overcome this inefficiency by increasing the number of available Wzx proteins present within the cell to perform low‐frequency translocation; however, the capacity for *wzx* overexpression in nature is poorly understood. In the absence of the triggering residues, the increase in number of Wzx can compensate for the reduced translocation efficiency (Hong & Reeves, 2014; Liu et al., 2015). The fact that Wzx overexpression alone can dramatically improve O‐antigen production implies that Wzx does not need to be part of a multi‐protein complex to function as a flippase. However, this does not necessarily infer that Wzx cannot behave differently if it is in a complex, as has been observed for the *E. coli* K‐12 ECA Wzx (Marolda, Tatar, Alaimo, Aebi, & Valvano, 2006).

The structural elements responsible for triggering high‐frequency translocation in Wzx_1_ and Wzx_2_ appear to be the terminal DDH or DDH‐like residues. The ability of both Wzx to flip several different DDH residues suggests that specific structural elements are directly recognized by Wzx. For instance, the different preferences of the two Wzx might be explained by Abe*p*, Tyv*p* and Par*p* all having a carbon‐6 positioned above the ring plane that is recognized by Wzx_2_, while the two‐carbon structure protruding from the furanose rings of Alt*f* and Par*f* may be recognized by Wzx_1_ (Figure 7). A possible trigger for Wzx_1_ translocation by Asc*p* is harder to identify, however, the different orientation of its carbon‐6 relative to Abe*p*, Tyv*p* and Par*p*, which cannot be flipped by Wzx_1_ (Table 3), may be important. Direct recognition of specific DDH structural elements may also explain the ability of Wzx_4_ and Wzx_8_ to flip the O:2a O unit with high efficiency. The cognate O:12 O unit of Wzx_8_ contains a terminal yersiniose (Yer(A)*p*), which is structurally identical to Abe*p* outside of a two‐carbon branched‐chain on carbon‐4 (Figure 7), so both residues may interact equally well with the Wzx_8_ recognition site. The terminal residues of the Wzx_4_, Wzx_5_ and Wzx_7_ cognate O units are all colitose (Fig. 7), yet Wzx_4_ was much more efficient at flipping the O:2a O unit. This may be due to its cognate O:6 O unit also having a Yer(A)*p* residue (Fig. S2) that may be recognized by Wzx_4_, thus facilitating recognition of the O:2a Abe*p* residue.

Despite the scarcity of closely related O‐antigen gene clusters and structures in most species, there is some supporting evidence for Structure‐specific Triggering being a broadly applicable model. The *S. enterica* O42 Wzx is known to be highly efficient in the translocation of the *E. coli* O1A O unit (Table 3), possibly due to its cognate O unit having the same terminal residue as O1A (Liu et al., 2017). The trisaccharide O units of *Pseudomonas aeruginosa* serotypes O2, O5, O16, O18, and O20 all share a common Wzx, but the only conserved sugar among the O units is the initial UndPP‐linked *N*‐acetylfucosamine (Islam & Lam, 2014). The remaining two residues on each O unit are both uronic acid sugars with only minor linkage variations, which may be the critical element required to trigger high‐frequency Wzx translocation. This example illustrates the importance of experimentally varying the O unit for studying Wzx specificity, which is necessary to prove a requirement for a specific trigger. Although additional functional and structural studies are still necessary, these known instances of diverse Wzx with both single‐residue specificity and a capacity to translocate a broader range of substrates suggest that our Structure‐Specific Triggering model does provide a foundation for resolving these seemingly disparate properties and developing a deeper understanding of the complex nature of Wzx‐substrate interactions.

## CONFLICT OF INTEREST

MAL and PRR contributed to the conception and design of the study. MAL and PM contributed to the acquisition, analysis and interpretation of the data. MAL and PRR contributed to the writing of the manuscript. The authors declare that they have no conflicts of interest.
