# Supplementary material for: Wzx flippases exhibiting complex O‐unit preferences require a new model for Wzx–substrate interactions
Source: Microbiologyopen. 2018 Jun 10;8(3):e00655. doi: 10.1002/mbo3.655 (PMC6436433; doi:10.1002/mbo3.655)

SUPPORTING INFORMATION

Fig. S1. Restoration of O-antigen production from O:1a, O:2a and O:4b O-antigen gene cluster clones containing deleted or alternative *wzx* genes. LPS samples were extracted from P6124 harbouring the indicated O-antigen gene cluster constructs and overexpressed *wzx* clones, and separated on a 13% (v/v) polyacrylamide gel by tricine-SDS-PAGE, followed by detection via silver staining. The location of LPS without O antigen (lipid A-core) is indicated.

Fig. S2. The *Y. pseudotuberculosis* O units translocated by the Wzx_3, Wzx_4, Wzx_5, Wzx_7 and Wzx_8 flippases examined in this study. O-unit structures are shown without their polymerisation linkages. O units are shown in boxes, with the relevant Wzx indicated below each box.

Fig. S3. Schematic for the assembly of the WT O:2a O-antigen gene cluster onto the Operon Assembly Vector (OAV) by yeast homologous recombination. The full-length O:2a O-antigen gene cluster from Fig. 1 is shown at the top of the figure. The green, red and purple bars represent the *Sal*I fragments from pPR1213 and pPR1214 and the ~4.4 kb *Hin*dIII fragment from pPR981, respectively. The position of each bar indicates the regions of the O:2a O-antigen gene cluster that they contain. The sites where homologous recombination occurs between the DNA fragments and/or the *Sma*I-digested OAV (shown below the bars) are indicated by the grey areas with dashed lines. The pink-coloured arrows on the OAV represent the *E. coli* K-12 *galF*, *gnd* and JUMPStart (JS) sequences, which flank the *Y. pseudotuberculosis ddhD* and *wzz* "hook" sequences (orange and blue arrows, respectively). The *CYH2* gene on the OAV (red arrow) is a counterselective cycloheximide-sensitive marker eliminated during homologous recombination with the DNA fragments, resulting in reversion of the yeast strain to cycloheximide resistance. Shown at the bottom of the figure inside the red box is the final arrangement of the O:2a O-antigen gene cluster in the OAV after homologous recombination, with the gene cluster now under the control of the JS region.

Fig. S4. Comparison of the LPS profiles of the O:2a O-antigen gene cluster cosmid (pPR981) and clone (pPR2272) expressed in SΦ874. LPS samples were extracted and separated on a 13% (v/v) polyacrylamide gel by tricine-SDS-PAGE, followed by detection via silver staining. The location of LPS without O antigen (lipid A-core) is indicated.

Fig. S1

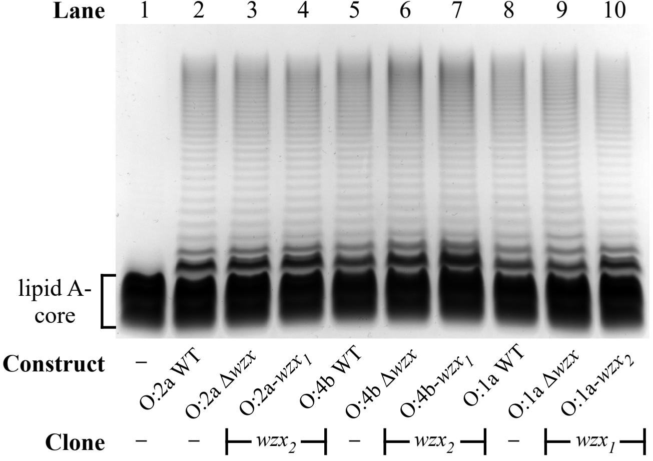

Lane 1 2 3 4 5 6 7 8 9 10
lipid A-core
Construct
–
O:2a WT
O:2a Δwzx
O:2a-wzx1
O:4b WT
O:4b Δwzx
O:4b-wzx1
O:1a WT
O:1a Δwzx
O:1a-wzx2
Clone
–
–
wzx2
–
wzx2
–
wzx1

Fig. S2

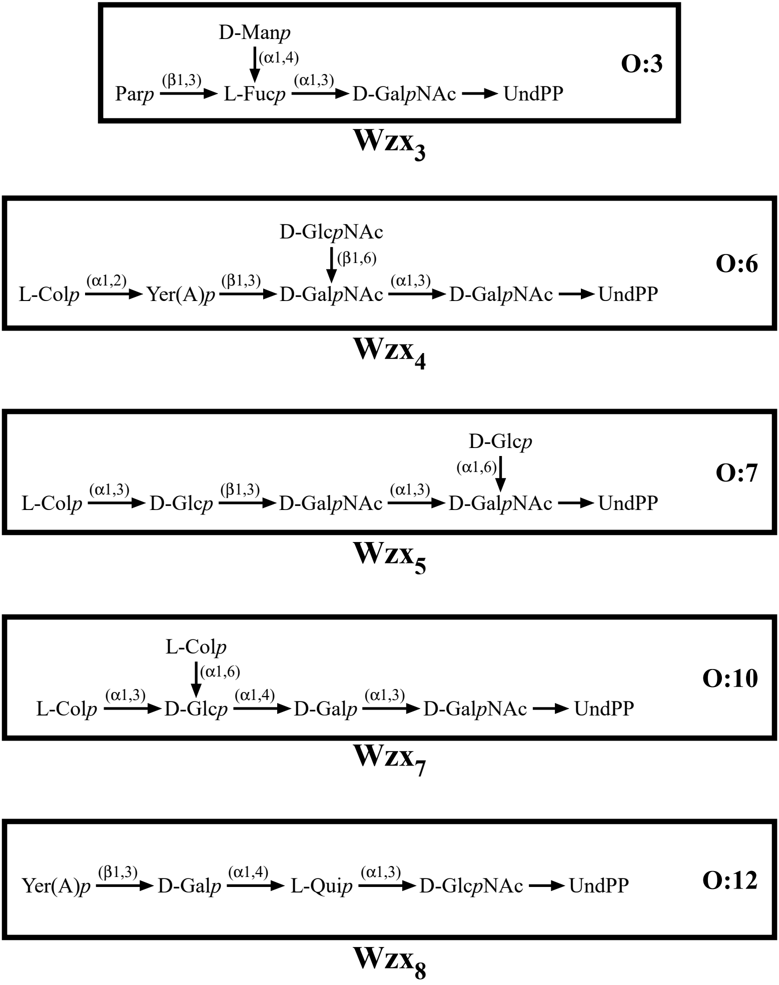
D-Manp
(α1,4)
Parp (β1,3) L-Fucp (α1,3) D-GalpNAc UndPP
O:3
Wzx3
D-GlcpNAc
(β1,6)
L-Colp (α1,2) Yer(A)p (β1,3) D-GalpNAc (α1,3) D-GalpNAc UndPP
O:6
Wzx4
D-Glcp
(α1,6)
L-Colp (α1,3) D-Glcp (β1,3) D-GalpNAc (α1,3) D-GalpNAc UndPP
O:7
Wzx5
L-Colp
(α1,6)
L-Colp (α1,3) D-Glcp (α1,4) D-Galp (α1,3) D-GalpNAc UndPP
O:10
Wzx7
Yer(A)p (β1,3) D-Galp (α1,4) L-Quip (α1,3) D-GlcpNAc UndPP
O:12
Wzx8

Fig. S3

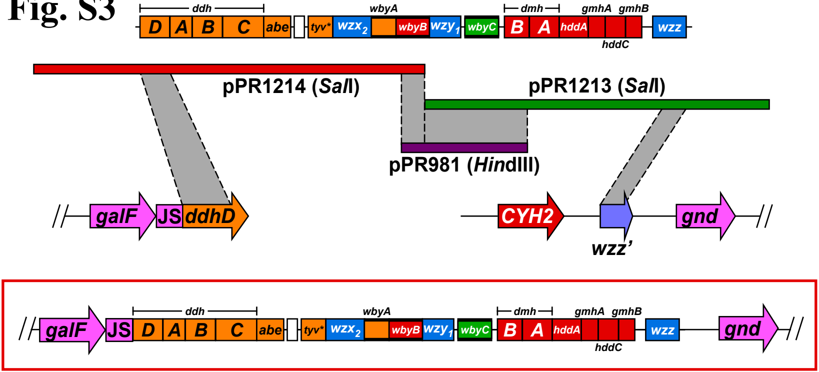

ddh
D
A
B
C
abe
wbyA
tyv*
wzx2
wbyB
wzy1
wbyC
dmh
B
A
hddA
gmhA
gmhB
hddC
wzz
pPR1214 (SalI)
pPR1213 (SalI)
pPR981 (HindIII)
galF
JS
ddhD
CYH2
wzz'
gnd

Fig. S4

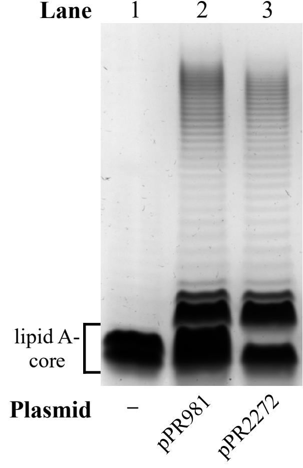

Supplement: Supplementary file 1 [file MBO3-8-e00655-s001.pdf]
