# Supplementary material for: Wzx flippases exhibiting complex O‐unit preferences require a new model for Wzx–substrate interactions
Source: Microbiologyopen. 2018 Jun 10;8(3):e00655. doi: 10.1002/mbo3.655 (PMC6436433; doi:10.1002/mbo3.655)
Supplement: Supplementary file 2 [file MBO3-8-e00655-s002.docx]

**Table S1:** Oligonucleotides used in this study

| Primer^[[1]](#endnote-1)^ | Sequence (5ʹ-3ʹ)^[[2]](#endnote-2)^ | Description^[[3]](#endnote-3)^ |
| --- | --- | --- |
| 5585F | ATGCTATGGTTATTTCATACCATAAGCCTAATGGAGCGAATT**gtgtaggctggagctgcttc** | PCR fragment with a *kan* cassette flanked by sequences for replacement of the SΦ874 *galE* gene |
| 5586R | ATGTGGATGATCAACGGGATTAAATTGCGTCATGGTCGTTCC*tagggataacagggtaat***TGGGAATTAGCCATGGTCC** |  |
| 6368F | GGAATTC*CCATGG*AA**ATGAGTAATATTAAAATA** | *wbyA* gene from *Y. pseudotuberculosis* O:2a strain M85 for cloning into the *Nco*I and *Bam*HI sites of pTrc99A |
| 6369R | GC*GGATCC***TTAATCTATAGAATTAAA** |  |
| 7201F | aaactgtttaaaatatagttctttagtggagtttaaaattaa**GTGTAGGCTGGAGCTGCTTC** | PCR fragment with an *rpsL-kan* cassette flanked by sequences for replacement of the O:2a *wzx_2_* gene |
| 7202R | gattatacgtaggaatacaaaatgatattttaatattactca**TCAGAAGAACTCGTCAAGAA** |  |
| 7203F | aaactgtttaaaatatagttctttagtggagtttaaaattaa**ATGAGAATAAATTATCAAATGGTATGG** | PCR fragment with the O:1a *wzx_1_* gene flanked by sequences for replacement of the O:2a *wzx_2_* gene |
| 7157R | GATTATACGTAGGAATACAAAATGATATTTTAATATTACTCA**TCACCTATCCTCTATATTATTTTTAATG** |  |
| 7204F | aaactgtttaaaatatagttctttagtggagtttaaaattaa**ATGCGTATTCCAACTCATCTTTTAA** | PCR fragment with the O:3 *wzx_3_* gene flanked by sequences for replacement of the O:2a *wzx_2_* gene |
| 7205R | gattatacgtaggaatacaaaatgatattttaatattactcaT**TAATAACTAGGATTATTAAGTACTTTCATT** |  |
| 7206F | aaactgtttaaaatatagttctttagtggagtttaaaattaa**ATGATTTTTAAAAATATAAATTATGATTTAAT** | PCR fragment with the O:6 *wzx_4_* gene flanked by sequences for replacement of the O:2a *wzx_2_* gene |
| 7207R | gattatacgtaggaatacaaaatgatattttaatattactcaT**TAATATTTAATATTATAATTCCAATAAAAATAG** |  |
| 7208F | aaactgtttaaaatatagttctttagtggagtttaaaattaa**TTGAAAATTAAATACTCTTTATTGATTGC** | PCR fragment with the O:10 *wzx_7_* gene flanked by sequences for replacement of the O:2a *wzx_2_* gene |
| 7209R | gattatacgtaggaatacaaaatgatattttaatattactca**TTACTGGCCACTGTTTTTTATAAAAT** |  |
| 7210F | aaactgtttaaaatatagttctttagtggagtttaaaattaa**ATGACTAAAATAAAATATTTAGCATTAAAG** | PCR fragment with the O:12 *wzx_8_* gene flanked by sequences for replacement of the O:2a *wzx_2_* gene |
| 7211R | gattatacgtaggaatacaaaatgatattttaatattactca**TTATAATTTTCTATTGTAATATTTTTTAAAAA** |  |
| 7228F | aaactgtttaaaatatagttctttagtggagtttaaaattaa**GTGAAGAATAGATTGATATTGAATAC** | PCR fragment with the O:7 *wzx_5_* gene flanked by sequences for replacement of the O:2 *wzx_2_* gene |
| 7229R | gattatacgtaggaatacaaaatgatattttaatattactcaT**TATTTTTTTATCTTTAGACCTATGAGAC** |  |
| 7258F | AGGA*CCATGG*CTA**TGCGGGTACCTACACATATAA** | *wzx_2_* gene from *Y. pseudotuberculosis* O:2a strain M85 for cloning into the *Nco*I and *Sal*I sites of pWQ552 |
| 7231R | AGTC*GTCGAC***TTACTCATCTAAACGTTTGTCCTT** |  |
| 7264F | AGGA*CCATGG*CT**ATGAGAATAAATTATCAAATGGTATGG** | *wzx_1_* gene from *Y. pseudotuberculosis* O:1a strain M444 for cloning into the *Nco*I and *Sal*I sites of pWQ552 |
| 7233R | AGTC*GTCGAC***TCACCTATCCTCTATATTATTTTTAATG** |  |
| 7234F | GTAA*GGATCC*T**ATGCGTATTCCAACTCATCTT** | *wzx_3_* gene from *Y. pseudotuberculosis* O:3 strain M451 for cloning into the *Bam*HI and *Sal*I sites of pWQ552 |
| 7235R | AGTC*GTCGAC***CTAATAACTAGGATTATTAAGTACTTTCATT** |  |
| 7236F | GTAA*GGATCC*T**ATGATTTTTAAAAATATAAATTATGATTTAAT** | *wzx_4_* gene from *Y. pseudotuberculosis* O:6 strain M460 for cloning into the *Bam*HI and *Sal*I sites of pWQ552 |
| 7237R | AGTC*GTCGAC***CTAATATTTAATATTATAATTCCAATAAAAATAG** |  |
| 7238F | GTAA*GGATCC*TA**TGAAGAATAGATTGATATTGAATACTAT** | *wzx_5_* gene from *Y. pseudotuberculosis* O:7 strain M462 for cloning into the *Bam*HI and *Sal*I sites of pWQ552 |
| 7239R | AGTC*GTCGAC***CTATTTTTTTATCTTTAGACCTATGAGA** |  |
| 7240F | GTAA*GGATCC*TA**TGAAAATTAAATACTCTTTATTGATTG** | *wzx_7_* gene from *Y. pseudotuberculosis* O:10 strain M2575 for cloning into the *Bam*HI and *Sal*I sites of pWQ552 |
| 7241R | AGTC*GTCGAC***TTACTGGCCACTGTTTTTTAT** |  |
| 7242F | GTAA*GGATCC*T**ATGACTAAAATAAAATATTTAGCATTAAAG** | *wzx_8_* gene from *Y. pseudotuberculosis* O:12 strain M2577 for cloning into the *Bam*HI and *Sal*I sites of pWQ552 |
| 7243R | AGTC*GTCGAC***TTATAATTTTCTATTGTAATATTTTTTAAAAA** |  |
| 7282F | taatctaacatggttggccagtgaatactggctaataataaa**GTGTAGGCTGGAGCTGCTTC** | PCR fragment with an *rpsL-kan* cassette flanked by sequences for replacement of the O:2a *abe-tyv** region |
| 7283R | ggtacccgcaattaattttaaactccactaaagaactatatt**TCAGAAGAACTCGTCAAGAA** |  |
| 7285F | taatctaacatggttggccagtgaatactggctaataataaa**ATGAAAATTCTTATTACCGGCGTTAG** | PCR fragment with the O:4b *prt-tyv* region flanked by sequences for replacement of the O:2a *abe-tyv** region |
| 7286R | ggtacccgcaattaattttaaactccactaaagaactatatt**TTAAACAGTTTCAACCCAATCAAGC** |  |
| 7300F | acagtagatagaatagttaaggacaaacgtttagatgagtaa**GTGTAGGCTGGAGCTGCTTC** | PCR fragment with an *rpsL-kan* cassette flanked by sequences for replacement of the O:2a *wbyA* gene |
| 7284R | atattcatttatttttcactctgaactttattattaattaat**TCAGAAGAACTCGTCAAGAA** |  |
| 7302F | acagtagatagaatagttaaggacaaacgtttagatgagtaa**TGAAAAGTAATATTATAATTGATAATAGTAACTC** | PCR fragment with the O:1a *wbyM* gene flanked by sequences for replacement of the O:2a *wbyA* gene |
| 7287R | atattcatttatttttcactctgaactttattattaattaat**TTAATGTAACCAAAAACTAAAATACTTACAAG** |  |
| 7289R | ttactattatcaattataatattacttttcataaaggtatta**TCAGAAGAACTCGTCAAGAA** | PCR fragment with an *rpsL-kan* cassette flanked by sequences for replacement of the O:1a *wzx_1_* gene |
| 7299R | ggtacccgcaattaattttaaactccactaaagaactatatt**TTAAATTGAACTATATCCACTAAGAAAAC** | PCR fragment with the O:1a *prt-wbyH* region flanked by sequences for replacement of the O:2a *abe-tyv** region (used with 7285) |
| 7301F | gttcaatttaaaatatagttctttagtggagtttaaaattaa**GTGTAGGCTGGAGCTGCTTC** | PCR fragment with an *rpsL-kan* cassette flanked by sequences for replacement of the O:2a *wzx_2_-wbyA* region on pPR2308 (used with 7284) |
| 7303F | gttcaatttaaaatatagttctttagtggagtttaaaattaa**ATGAGAATAAATTATCAAATGGTATGG** | PCR fragment with the O:1a *wzx_1_-wbyM* region flanked by sequences for replacement of the O:2a *wzx_2_-wbyA* region (used with 7287) |
| 7310F | aatagtgcaattaaagatattttacataaggaatataaatga**GTGTAGGCTGGAGCTGCTTC** | PCR fragment with an *rpsL-kan* cassette flanked by sequences for replacement of the O:4b *tyv* gene on pPR2313 |
| 7312R | tttattctcatttaattttaaactccactaaagaactatatt**TCAGAAGAACTCGTCAAGAA** |  |
| 7311F | aatagtgcaattaaagatacactaaacagaatgcgggtctga**GTGTAGGCTGGAGCTGCTTC** | PCR fragment with an *rpsL-kan* cassette flanked by sequences for replacement of the O:1a *wbyH* gene on pPR2314 |
| 7315F | AGTTC*GAATTC***ATGAAAATTCTTATTACCGGCG** | *prt-tyv* genes from *Y. pseudotuberculosis* O:4b strain M454 for cloning into the *Eco*RI and *Bam*HI sites of pTrc99A |
| 7316R | GTAA*GGATCC***TTAAACAGTTTCAACCCAATCAAG** |  |
| 7318R | GTAA*GGATCC***TTAAATTGAACTATATCCACTAAGAA** | *prt-wbyH* genes from *Y. pseudotuberculosis* O:1a strain M444 for cloning into the *Eco*RI and *Bam*HI sites of pTrc99A (used with 7315) |

1. Orientation of primers relative to their target gene(s) is indicated as: F, forward; R, reverse. [↑](#endnote-ref-1)
2. Primer sequences: **bold**, priming site for PCR amplification; *italicised and underlined*, restriction endonuclease recognition sequence; all remaining sequences are dummy sequences for restriction endonuclease digestion prior to cloning or to place a gene’s coding sequence in-frame with a vector’s internal start codon. [↑](#endnote-ref-2)
3. Oligonucleotides grouped together in the table represent primer pairs for PCR-amplification of the gene(s) indicated in the description. [↑](#endnote-ref-3)
